# Chylothorax after esophagectomy treated with inguinal intranodal lymphangiography and transvenous retrograde thoracic duct embolization

**DOI:** 10.1007/s12328-021-01429-z

**Published:** 2021-05-11

**Authors:** Yuta Sato, Yoshihiro Tanaka, Takeharu Imai, Hiroshi Kawada, Naoki Okumura, Nobuhisa Matsuhashi, Takao Takahashi, Masayuki Matsuo, Kazuhiro Yoshida

**Affiliations:** 1 Department of Surgical Oncology, Gifu university, graduate school of Medicine, 1-1 Yanagido, Gifu city, 501-1194 Japan; 2 Department of Radiology, Gifu University Hospital, Gifu, Japan

**Keywords:** Thoracic duct embolization, Lymphangiography, Esophageal cancer, Chylothorax, Chyle leaks

## Abstract

Chylothorax after esophagectomy is a serious complication that is associated with major morbidity due to dehydration and malnutrition. Reoperation with ligation of the thoracic duct is considered for patients with high-output chyle leaks that have failed conservative management. In this report, we present the treatment options for chylothorax after esophagectomy: inguinal intranodal lymphangiography and transvenous retrograde thoracic duct embolization. A 74-year-old man with esophageal cancer had been operated with thoracoscopic esophagectomy. Six days after surgery, he presented with high-output chyle leaks. Conservative treatment did not result in a significant improvement. Inguinal intranodal lymphangiography and transvenous retrograde thoracic duct embolization were performed 13 days after surgery and were technically and clinically successful. Inguinal intranodal lymphangiography and transvenous retrograde thoracic duct embolization are an effective treatment option, especially for patients after esophagectomy with reconstruction performed via the posterior mediastinal route, without the potential for damage the gastric tube and omentum.

## Introduction

Chylothorax is a severe complication that can occur after esophagectomy [1]. Surgical thoracic duct ligation is recommended for patients with high-output chylothorax (> 1000 ml/day) who have failed conservative treatment [2]. Thoracic duct embolization (TDE) has recently become a minimally invasive alternative to surgical thoracic duct ligation due to its efficacy [2–4]. In this report, we present a case of chylothorax treated with inguinal intranodal lymphangiography and transvenous retrograde TDE. The transvenous retrograde access for TDE may avoid possible injuries of intraperitoneal organs and structure, this method can be a safe treatment option for chylothorax before undergoing reoperation.

## Case report

A 74-year-old man with esophageal cancer after thoracoscopic esophagectomy had high-output chylothorax from day 6 post-surgery. We performed total parenteral nutrition, right-side pleural drainage and continuous intervenous injection of Octreotide (150 µg/day) from day 7 post-surgery, but were unsuccessful. Since the amount of chylothorax (> 4000 ml/day) was very high and the albumin levels in the blood (1.2 g/dl) did not improve, we considered reoperation, but before that, we selected inguinal intranodal lymphangiography on day 13 post-surgery.

**Fig. 1:**
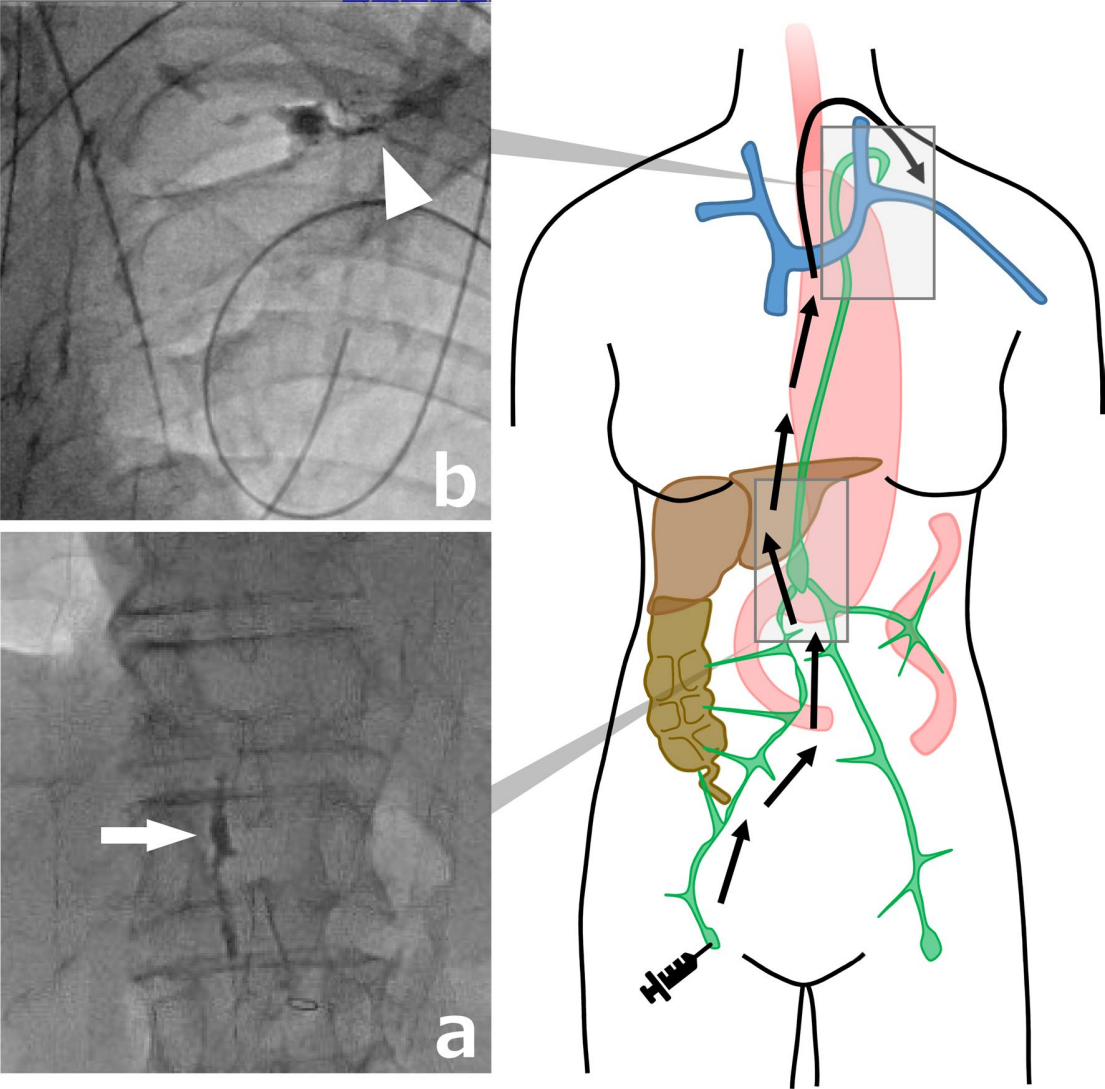
Fluoroscopic image of the presented case after inguinal intranodal lymphangiography. **a** Cisterna chyli at the L1 vertebral level (white arrow). **b** Lipiodol propagated into the left venous angle (white arrowhead)

First, inguinal intranodal lymphangiography was performed using a 22-gauge cathelin needle (Fig. 1a, b).

Puncture the superficial inguinal lymph node under ultrasound guidance and position the tip of the needle at the nodal hilum. Iodized oil (Lipiodol; Guerbet Japan, Tokyo, Japan) was gently injected manually at a rate of approximately 1–2 ml every 5 min under intermittent fluoroscopy. A total of 6 ml of Lipiodol was injected to opacify the lymphatic system up to the cisterna chyli, which was at the level of Th12/L1 disc.

**Fig. 2:**
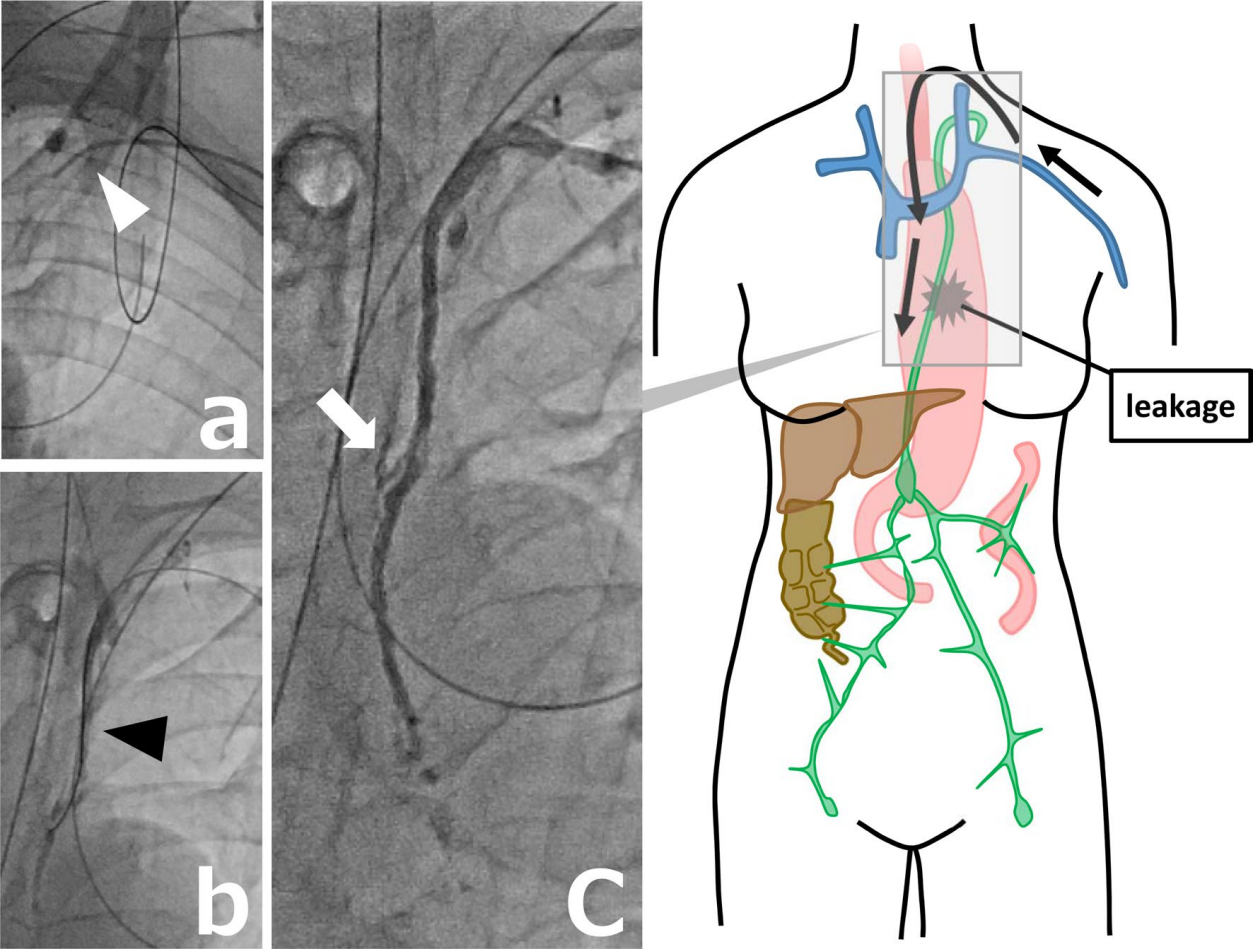
Transvenous retrograde thoracic duct embolization. **a**, **b** Microcatheter (white arrowhead) and microguidewire (black arrowhead) inserted in the thoracic duct from the left brachial vein to the junction of the thoracic duct with the vein. **c** Contrast is injected into the thoracic duct to confirm proper entry into the duct and to verify the site of leakage (white arrow)

**Fig. 3:**
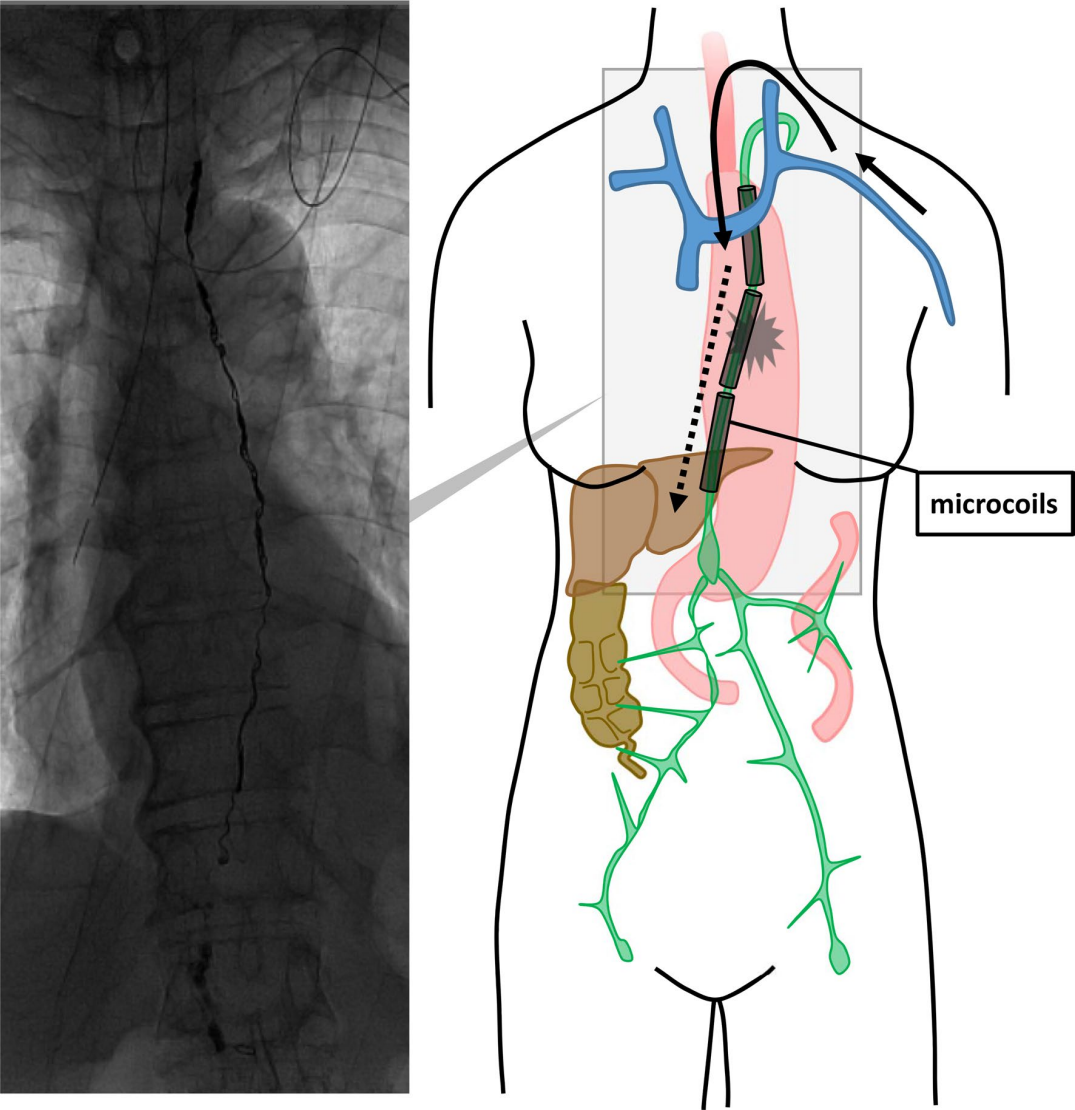
Situation after thoracic duct embolization with microcoils placed from directly above the cisterna chyli up to the distal thoracic duct

Although the leak could not be identified, since Lipiodol was discharged into a vein at the left venous angle, we decided to perform transvenous retrograde thoracic duct embolization (Fig. 2a, b, c). A 4-F introducer sheath (Super Sheath; Medikit, Tokyo, Japan) was inserted in the left brachial vein. In the approach from the left brachial vein to the junction of the thoracic duct with the vein (JTV), a 4-F cobra-shaped catheter (ANGIOMASTER; Terumo, Tokyo, Japan) and a 1.9-F microcatheter (Prograteλ19; Terumo) were used to access the thoracic duct. A 0.014-in. microguidewire (ASAHI CHIKAI; Asahi Intecc, Aichi, Japan) and a 0.016-in. microguidewire (ASAHI Meister; Asahi Intecc) were used to seek the JTV and guide the microcatheter. A small amount of contrast was injected to confirm proper entry into the thoracic duct and also to verify the site of leakage. Iodinated water-soluble contrast medium (iopamidol, Iopamiron 370 Injection syringe; BAYER, Osaka, Japan) was used in all transcatheter contrast radiography. The leak could be accurately identified by being able to entry into the thoracic duct. Once the site of the leak was identified, the microguidewire and microcatheter were inserted beyond the suspected site of leakage and placed in the cisterna chyli, and microcoils were used to embolize the thoracic duct. The microcoils, including Target XXL 360 Detachable Coils and Target 360 soft (Stryker, Kalamazoo, MI) and Tornado Embolization Coil 4/2 mm and 3/2 mm (Cook Medical, Bloomington, IN) were placed from near the cisterna chyli to the cephalic side of the aortic arch (Fig. 3). The time for all these procedures was 260 min. This procedure cost 29,000 Japanese yen. Although N-butyl-2-cyanoacrylate (NBCA) may be used in combination, it is often difficult to reuse the catheter, so only microcoils were placed.

**Fig. 4:**
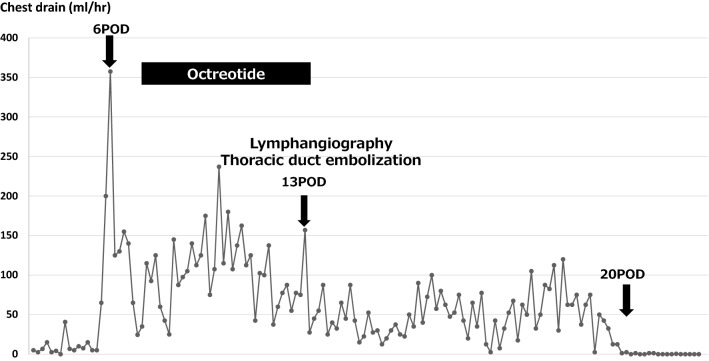
The changes in chylous discharge before and after the treatment

Chyle output gradually decreased after TDE and stopped completely on day 20 post-surgery. The patient’s post-interventional course was uneventful (Fig. 4).

## Discussion

Chylothorax is a severe complication after esophagectomy. Patients with chylothorax are usually treated with conservative treatment options (total parental nutrition, low fat diet, octreotide and pleural drainage). Historically, surgical thoracic duct ligation is recommended for patients with high-output chylothorax (> 1000 ml/day) who have failed conservative treatments [2]. TDE has recently become a minimally invasive alternative to surgical thoracic duct ligation due to its efficacy.

Catheter cannulation to the thoracic duct is necessary for diagnosis of chylothorax and TDE, and various approaches have been reported [5]. Transabdominal antegrade access is a common access method reported by Cope et al. [6]. This method is established by needle puncture and subsequent catheterization of the thoracic duct by the microcatheter that traverses various organs along its way. Thus, this access carries the risk of peritoneal organ penetration including arteries and the intestines [7]. In reconstruction performed via the posterior mediastinum after esophagectomy, the gastric tube and the omentum, which includes feeding vessels, runs so as to contact the cisterna chyli and the lower thoracic duct. It is reported that the transabdominal antegrade access route passes through the diaphragmatic crus in 71.4% of cases [8], the omentum feeding the gastric tube may have to be transgressed multiple times. Damage to the feeding blood vessels and hematoma formation due to omental puncture may lead to a decrease in blood flow in the anastomosis and cause leakage.

The benefit of transvenous retrograde access does not require puncture of the organs, but the problem is that the success rate is not high. In particular, it is difficult to insert the microcatheter retrogradely into JTV, with a reported success rate of 61.5% [9]. In the JTV, there are many branches that join from the cervical region or arms. The cervical part of thoracic duct is reported to have a plexiform configuration without a prominent main duct in 26% of cases [10]. In this type, the narrow thoracic ducts and complex branching make it difficult for the retrograde advancement of microcatheter through a valve at the JTV, which can damage the valve [9].

**Fig. 5:**
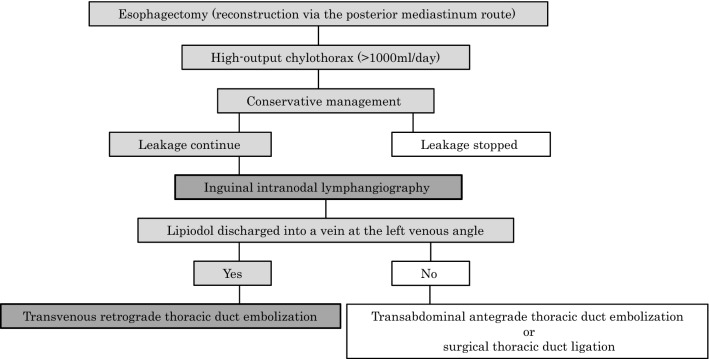
The flowchart for chylothorax treatment that we propose

Although transvenous retrograde TDE is not widely reported and has a low success rate, it can be a new treatment option with the advantage of avoiding damage to the gastric tube and omentum. Inguinal intranodal lymphangiography is suggested for patients with high-output chylothorax (> 1000 ml/day) after esophagectomy who have failed conservative management, especially when reconstruction is performed via the posterior mediastinal route. Transvenous retrograde TDE may be selected as a treatment before surgical thoracic duct ligation, if Lipiodol would discharge into a vein at the left venous angle (Fig. 5).

## Data Availability

Not applicable.
